# Quantitative Support for the Metabolic Load Hypothesis: Metabolic Rate Measures Reveal Host‐Dependent Growth Costs in a Polyphagous Herbivore

**DOI:** 10.1002/ece3.72509

**Published:** 2025-12-11

**Authors:** Katharina Schneider, Kevin T. Roberts, Philipp Lehmann, Christopher W. Wheat, Niklas Janz, Sören Nylin

**Affiliations:** ^1^ Department of Zoology Stockholm University Stockholm Sweden; ^2^ Department of Animal Physiology, Zoological Institute and Museum University of Greifswald Greifswald Germany

**Keywords:** ecological specialization, energy allocation, insect‐plant interaction, metabolic load hypothesis, metabolic rate

## Abstract

The interactions between phytophagous insects and their host plants show a strong trend toward specialization. However, the reasons behind this trend remain largely unclear, at both the evolutionary and mechanistic level. One possible explanation is an increased energy expenditure for digesting and metabolizing more challenging hosts included in a broader host repertoire, which may reduce the energy available for other processes such as growth and development (“metabolic load hypothesis”). Differences in the performance across various hosts could reflect such costs. Using the polyphagous *Polygonia c‐album* (comma butterfly), we tested whether observed performance differences can be linked to variation in the energetic requirements. For this, we measured the metabolic rate of larvae feeding on three different host plants and converted it into the amount of CO_2_ produced per gram of mass gain (“growth cost”) to assess how much energy is allocated to growth vs. digestion and assimilation. The metabolic rate of larvae feeding on a chemically more challenging plant ( *Ribes uva‐crispa* ) was similar to that of individuals on the host supporting the highest growth rate ( *Urtica dioica* ). However, larvae on *Ribes uva‐crispa* exhibited a higher energy demand per unit of growth and a lower growth rate, indicating a different energy allocation in growing larvae than when they were reared on a chemically less challenging plant. Our findings suggest that energy expenditure for digesting different hosts varies and can have direct consequences for larval performance. This indicates that the trend toward ecological specialization may, at least partly, be driven by selection to reduce the energetic costs for detoxification and digestion, in support of the metabolic load hypothesis.

## Graphical abstract

The metabolic load hypothesis was tested in *Polygonia c‐album* caterpillars by comparing metabolic costs across three host plants. Larvae feeding on a chemically challenging host showed higher energy demands per unit of growth and reduced performance. These findings suggest that variation in the energetic requirements among host plants may underlie differences in growth rates and could promote ecological specialization.

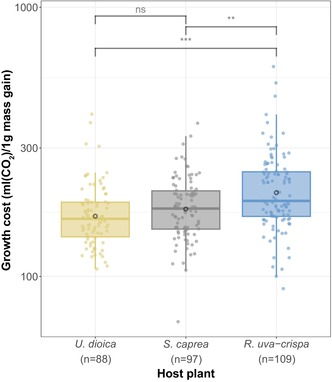

## 1 Introduction

Insects feeding on plants represent one of the most prominent species interactions on the planet. Of all the phytophagous insects, 80%–90% are assumed to be specialized to feeding on a narrow diet comprising three or fewer plant families and sometimes even only one species (Schoonhoven et al. 2005; Forister et al. 2015). The reason for this trend toward ecological specialization is however still not fully understood (see review by Hardy et al. 2020). One potential explanation that was proposed already by Whittaker and Feeny (1971) and Krieger et al. (1971) is an increased energetic cost for the digestion of different (harmful) plant components associated with a broader host repertoire (the “metabolic load hypothesis”; Appel and Martin 1992). Although evidence for such costs is currently rare (but see Cresswell et al. 1992), it is often assumed that species with a narrow diet are more efficient in not only the detoxification of chemical defenses (Cornell and Hawkins 2003; Li et al. 2004) but also in the overall processing of their hosts, due to encountering fewer and less diverse substances. A recent meta‐analysis gave some support for the prediction from this hypothesis that specialists should grow faster than generalists on shared hosts (Rothwell and Holeski 2020), but the mechanisms behind such patterns are in need of further investigation.

One promising approach to address this question is to investigate the efficiency of host use in the context of the energetic demands of processing one or several host plants. The digestion of food comprises several steps in which ingested material is mechanically and (bio)chemically broken into smaller compounds. The processes involved in the catabolism of food particles and consequently the absorption and assimilation of nutrients require energy (Karasov and del Martinez Rio 2007). The exact amount of required energy, however, depends on several factors, like temperature, body mass, meal size (Secor and Faulkner 2002; Khan et al. 2015; McCue et al. 2016) and the composition and quality of the meal (see e.g., McCue et al. 2005; Henriksen et al. 2015; Clark et al. 2016). Besides, the digestion of further compounds, like defensive chemicals, can sometimes demand specific pathways and processes, which might be associated with additional costs. An increased energetic investment into the digestion of specific food or molecules can consequently mean that this energy is no longer available for other processes like growth, development and reproduction (Karban and Agrawal 2002). Such trade‐offs should become especially apparent in species that can feed on a variety of hosts, as they are exposed to food sources that can differ greatly in their morphological but also chemical composition (nutrients and secondary metabolites). This diversity could be expected to result in different energetic costs for the digestion of various diets.

Growth rates of phytophagous insects typically differ depending on the host plant they are feeding on (e.g., Nylin and Gotthard 1998; Ajmal et al. 2024; El‐Refaie et al. 2024). Such host‐specific differences in performance could potentially—at least partially—be attributed to variations in the energetic demands during digestion. Studies focusing on the transcriptional profiles in response to different host plants provide support for the involvement of very diet‐specific pathways associated with metabolism, detoxification and stress response (Celorio‐Mancera et al. 2013, 2023; Birnbaum and Abbot 2020; Schneider et al. 2024). The extent of transcriptional changes moreover differs depending on host plant (Schneider et al. 2024), further suggesting that some plants might represent a more challenging environment and are costlier to digest and metabolize. While host‐specific metabolic loads have been debated, and evidence in support is as yet rare (Cresswell et al. 1992) compared to studies rejecting its effect on performance (Neal 1987; Van Loon 1993; Castañeda et al. 2009; Bastias et al. 2017), it has to be noted that these studies primarily tested the metabolic load hypothesis in reference to chemical plant defenses. Although originally proposed in the context of such allelochemicals, it needs to be considered that also other features and components of a host plant could cause additional energetic costs.

The energetic cost of a meal can be assessed by evaluating the metabolic rate after food intake. In general, the metabolic rate describes the overall amount of energy an organism needs under specific conditions and within a given unit of time (e.g., Treberg et al. 2016). Under resting conditions, without food consumption or digestion, this rate describes the minimal amount of energy that is required for maintenance and survival (Standard metabolic rate, Treberg et al. 2016). Activities like feeding, digesting and locomotion will result in a higher energetic expenditure, and consequently a higher metabolic rate (Nespolo et al. 2005). To get a clear understanding of how much of the consumed energy can be associated with digestion and subsequent catabolic metabolism of a host (specific dynamic action; McCue 2006; Secor 2009), it is, however, necessary to also include an estimate for the allocation of energy into different processes. A commonly accepted method for this is the efficiency of the conversion of digested food into body mass (conversion efficiency of digested food; Waldbauer 1968). In most studies this efficiency is based on the ratio between the mass gain and the differences between consumed and excreted material (see e.g., Neal 1987; Cresswell et al. 1992; Van Loon 1993). As excreted material (frass) can also contain urine, intestinal secretions and cellular debris of the digestive tract (Waldbauer 1968), this difference is only an approximate estimate of the actual amount of digested food. Additionally, it does not account for the actual energetic costs of growing on a particular plant. We propose that a more reliable approach for describing the energy allocation would be to quantify the energetic demand per unit mass gain based on the metabolic rate. At a constant temperature and with no activities other than those related to digestion, any differences in the postprandial metabolic rate (i.e., the metabolic rate after a meal) between food treatments will be indicative of the associated diet‐dependent processes.

Polyphagous insects represent valuable models for examining how performance differences across multiple diets correlate with underlying molecular and physiological processes. *Polygonia c‐album* (Nymphalidae; Linnaeus 1758), for instance, is characterized by an extraordinary host repertoire for a butterfly, as the larvae are specialized to feed on a few species within multiple different families of plants: Urticaceae (*Urtica*), Cannabacaea (*Humulus*), Ulmacaeae (*Ulmus*), Salicaceae (*Salix*), Grossulariaceae (*Ribes*) and Betulaceae (*Betula*, *Corylus*; Nylin 1988). These host plants not only differ in their growth form (trees, bushes and herbs) and evolutionary divergence, they also vary in their chemical properties: *Urtica dioica*, for instance, is rich in nitrogen, and characterized by a high mineral content (notably phosphorus, calcium, magnesium and potassium; Taylor 2009) and a relatively high N/C ratio (0.06–0.13; Wedell et al. unpublished data). Its water content varies seasonally between 72%–80% (Wedell et al. unpublished data). Furthermore, the leaves contain flavonoids, alkaloids, phenolic acids (quercetin, caffeic, p‐coumarin and ferulic acids) as well as coumarins (scopoletin), saponins and tannins (Hegnauer 1973; Abdeltawab et al. 2012). Studies on transcriptional responses confirmed that genes involved in transmembrane transport (Synaptic vesicle glycoprotein‐like, Alkaline phosphatase), among others for nitrate (Sialin‐like) were indeed upregulated in larvae feeding on *U. dioica* . Genes involved in overcoming chemical defenses of plants and immune response (Gloverin‐like protein, N‐acetylmuramoyl‐L‐alanine amidase, Trypsin‐like serine protease) were also upregulated (Celorio‐Mancera et al. 2023; Schneider et al. 2024).

*Salix caprea* leaves contain a complex array of secondary metabolites predominantly including benzoic and cinnamic acids, flavonoids, tannins and salicinoids (Hegnauer 1973; Hallgren et al. 2003). Young leaves are particularly rich in sugars and elevated contents of minerals such as zinc, iron and manganese have been reported (Hegnauer 1973). The N/C ratio (0.05–0.08) and moderate water content (55%–72%) are comparable to other woody plants (Wedell et al. unpublished data). Larvae feeding on *S. caprea* showed an upregulation of genes involved in signaling, ion transport and responses to chemical stress or other stimuli (Schneider et al. 2024), suggesting that not only specific toxins and other defense components (tannins, flavonoids; Hegnauer 1973; Hallgren et al. 2003), but also the overall composition of a plant, may require specific molecular processes for digestion.

Leaves of *Ribes uva‐crispa* possess flavonoids (quercetin, kaempferol, catechin), hydroxycinnamic acids and saponins (Hegnauer 1973). They also contain notable levels of ascorbic acid and simple sugars such as Hamamelose (Hegnauer 1973). Larvae of *P. c‐album* that fed on *R. uva‐crispa* have repeatedly been reported to show upregulation of genes involved in response to oxidative stress and detoxification (Spermine oxidase, Glutathione S‐transferase, and Serine proteases; Celorio‐Mancera et al. 2023; Schneider et al. 2024). Additionally, genes associated with the strengthening and repair of the peritrophic membrane, a physiological barrier to harmful plant compounds, were upregulated (Celorio‐Mancera et al. 2013, 2023; Schneider et al. 2024). This could represent a reaction to the cyanogenic glucosides found in the leaves of *R. uva‐crispa* and related *Ribes* species (Hegnauer 1973; Bjarnholt and Møller 2008).

Such differences in the chemical composition could indicate distinct energetic demands during the digestion of those host plants. Given this body of literature, here we use larvae of *P. c‐album* as a model system to test whether increased energetic costs associated with the chemical properties of the food plants can explain a reduced performance (i.e., growth rate). We predict that:

1. Larval performance will differ between host plants. Supporting this hypothesis are previously reported hierarchy patterns (Nylin 1988; Nylin and Janz 1993; Janz et al. 1994, 2009) which showed that individuals perform better on *U. dioica* compared to *S. caprea* . Individuals reared on *R. uva‐crispa* are expected to show the lowest performance (Nylin 1988; Janz et al. 2009).
2. The overall metabolic rate of larvae will differ between diets. If the use of all host plants had the same energetic costs, no differences in the metabolic rates should be found between various diets. However, based on the observed performance hierarchies described above, we posit that the energetic costs on different host plants are not equal.
3. Variation in performance is the result of different growth costs. While the metabolic rate can give a good estimate for the amount of energy associated with the use of a specific host, it cannot distinguish between the energy costs for digestion versus the ones for other processes (e.g., growth). To further disentangle the energy expenditure of different processes, the amount of energy (metabolic rate) that is invested into growth ('growth cost') on a particular host, was calculated. As individuals that fed on *U. dioica* repeatedly showed high growth rates, it is expected that the amount of energy required for one growth unit is comparatively low. In contrast, the lower growth rates measured on *R. uva‐crispa* could suggest a reduced energy allocation for growth, due to increased investment into other processes.

## 2 Materials and Methods

### 2.1 Butterfly Collection and Rearing

Mated females were caught in Åkersberga (N 59°28′56″, E 18°21′18″) and Hansta (N 59°26′11″, E 17°54′14″) close to Stockholm, Sweden (*n* = 15) in spring 2022. The collected females were brought into the laboratory and put into separate cages (50 × 50 × 40 cm) containing a cut branch of *Urtica dioica* (stinging nettle). Each cage was placed below a light source (Solar Raptor, L:D 8:16, with 25°C during the day and 18°C during dark conditions), and had a transparent roof and a net in the front. The bottoms of the cages were covered with wet paper towels. Sugar water was provided for feeding. Eggs were collected by hand every 24 h and moved to 17°C (L:D 12:12).

Upon hatching, larvae were moved into individual cups (volume: 1.25 oz) with each containing a leaf of either *U. dioica* , *Salix caprea* (sallow), or *Ribes uva‐crispa* (gooseberry), that were collected in the surroundings of Stockholm University, Stockholm, Sweden (N 59°21′47″, E 18°03′3″; leaves were taken from multiple plant individuals when possible). For this, it was ensured that larvae of different families were evenly distributed among the host plants (split brood design).

To avoid drying of the leaves, wet cotton was wrapped around the petiole. Leaves from *S. caprea* were additionally put on a wetted piece of sponge cloth. Leaf quality and amount were checked daily and, if necessary, replaced by fresh material.

Larvae were kept at 22°C (L:D 12:12). Cups were moved randomly every day to avoid potential position effects.

### 2.2 Performance Assessment

Larval performance was measured by calculating the growth rate on different host plants. Larvae were weighed in the 4^th^ instar. To avoid negative growth rates, larval mass was converted into milligrams. The growth rate was then calculated using the logged mass and the developmental time from hatching until reaching the 4^th^ instar:

$$\text{Growth rate}=\log\left(\text{Larval mass}\times 1000\right)/\text{Developmental time in days}.$$

### 2.3 Respirometry Measurements

Metabolic rate was measured to quantify digestion‐related energy consumption at three different developmental stages: 3^rd^ instar, 4^th^ instar and pupae, using a syringe flow respirometry approach following the protocol of Roberts et al. (2021) and Roberts and Williams (2022). For this, larvae that had just molted into the respective instar first got a fresh leaf. After 3–5 h the cups were checked again. Larvae that had fed were weighed and transferred into a 20 mL plastic syringe. Direct measurement of food intake was not feasible for this study, so visual assessment was used to standardize the consumption across individual larvae. For a subset of the individuals we also measured the metabolic rate at the pupal stage, 1–4 days after pupation.

The syringes containing a larva or pupa were first flushed with air from which H_2_O and CO_2_ were removed with Drierite (Sigma‐Aldrich) and Ascarite (Thermo Fisher Scientific) columns. A syringe not containing any individuals was added as a control. Afterwards the syringes were incubated for one hour at 22°C in the dark. Incubation in the dark was necessary to avoid additional activity like moving (personal observations). This should ensure that the produced CO_2_ was only associated with processes involved in maintenance, digestion, metabolism and growth. To measure the amount of CO_2_ produced during this incubation, 7 (3^rd^ instars) or 10 mL (4^th^ instars and pupae) of the incubated air were injected and first went through a magnesium perchlorate column (Sigma Aldrich) to remove H_2_O produced by respiration. As a reference we also measured the CO_2_ for a few minutes before injecting the first sample. CO_2_ was then measured with a CA‐10 carbon dioxide analyzer (Sable Systems International). The CO_2_ content of the empty syringe was included as a control. Measurements were taken at room temperature (around 20°C). After the metabolic rate was measured, larvae were transferred back to their cup with a fresh leaf of the respective host plant.

The amount of CO_2_ respired by each larva or pupa within an hour was determined following the calculations in Ittonen et al. (2023). In short, first the CO_2_ content of the reference was removed from each measurement. Baseline‐corrected values were converted into air fractions and multiplied by the flow rate in Expedata (version 1.9.10; Sable Systems). The resulting values were integrated over time. The CO_2_ content of the control syringe was subtracted and the values were corrected by the total syringe volume divided by the injected volume. The total volume was subsequently divided by the time the individuals spent in the syringe giving the production rate in milliliters per minute ($\dot{V}{\mathrm{CO}}_{2}$).

### 2.4 Analysis

Our study compared the performance and metabolic rate of larvae of *P. c‐album* when reared on different host plants (Table 1).

**TABLE 1:** Replication statement.

Scale of inference	Experiment	Developmental stage	Host plant/factor level	Replicates
Individuals	Performance	4^th^ larval instar	*Urtica dioica*	88
			*Salix caprea*	97
			*Ribes uva‐crispa*	109
	Metabolic rate	3^rd^ larval instar	*Urtica dioica*	49
			*Salix caprea*	52
			*Ribes uva‐crispa*	56
		4^th^ larval instar	*Urtica dioica*	88
			*Salix caprea*	97
			*Ribes uva‐crispa*	109
		Pupa	*Urtica dioica*	18
			*Salix caprea*	21
			*Ribes uva‐crispa*	29
	Growth cost	4^th^ larval instar	*Urtica dioica*	88
			*Salix caprea*	97
			*Ribes uva‐crispa*	109

*Note:* The number of individuals per host plant is given for every experiment.

The effect of diet on larval performance and metabolic rate was tested with generalized linear mixed models using the package glmmTMB (version 1.1.9, Brooks et al. 2017). For this, we first tested for an effect on the growth rate, with host plant as a fixed effect. Family ID was included as a random effect:

$$\text{Growth rate}\sim \text{Host plant}+\left(1|\;\text{Family}\;\mathrm{ID}\right),\text{family}=\text{Gaussian}.$$

To estimate the mass‐scaling relationship of the metabolic rate, we tested the effect of log‐transformed larval mass and host plant on the log‐transformed CO_2_ production rate ($\dot{V}{\mathrm{CO}}_{2}$). First, we assessed the significance of the interaction between body mass and host plant using the package lmtest (version 0.9.40, Zeileis and Hothorn 2002). As the interaction was not significant (likelihood ratio test: χ^2^ = 2.34, df = 2, *p* = 0.31) and did not improve model fit, it was removed from the final model, resulting in a common mass‐scaling exponent. Note that host‐specific differences in mass‐scaling were assessed only in the larval stage.

$$\log\left(\dot{\text{V}}{\mathrm{CO}}_{2}\right)\sim \log\left(\text{Larval mass}\right)+\text{Host plant},\text{family}=\text{Gaussian}$$

To test for diet‐dependent differences in the larval metabolic rate, we measured the effect of plant in interaction with developmental stage on the amount of CO_2_ produced within 1 h (production rate, 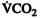). Logged larval mass was included as a covariate in the models. Furthermore, Individual ID nested in Family ID was included as a random effect. To test for a host effect on the metabolic rate in pupae, a similar model without developmental stage and Individual ID was used.

$$\begin{array}{c} \dot{\text{V}}{\mathrm{CO}}_{2}\sim \text{Host plant}\times \text{Developmental stage}+\log\left(\text{Larval mass}\right)\\ +\left(1|\;\text{Family}\;\mathrm{ID}/\text{Individual}\;\mathrm{ID}\right),\text{family}=\text{Gaussian} \end{array}$$

To assess how much of the consumed energy was actually invested into growth, we calculated the amount of CO_2_ (in mL) that is produced per hour by 1 g of mass gain in 4^th^ instar larvae:

$$\text{Growth cost}=\left(\text{Developmental time}/\text{Larval mass}\right)\times 24\times 60\times \dot{\text{V}}{\mathrm{CO}}_{2}.$$

Here a lower value would indicate that less energy is necessary to achieve an increase in mass.

The effect of diet on the growth cost was then tested using the host plant as a fixed effect. Here, Family ID was again included as a random effect. Since we had information about the sex for some of the individuals, we also included sex as another covariate in the initial model. However, since its effect was non‐significant (χ^2^ = 2.4908, df = 2, *p* = 0.2878), sex was excluded from the final model:

$$\text{Growth cost}\sim \text{Host plant}+\left(1|\;\text{Family}\;\mathrm{ID}\right),\text{family}=\text{Gaussian}.$$

Model fit of all models was checked using DHARMa (version 0.4.6, Hartig 2022). The Anova function from the R package car (version 3.1–2; Fox and Weisberg 2019) was used to assess significance. Post hoc tests were done with emmeans (version 1.8.2, Lenth 2022) using Tukey adjustment for multiple testing. All data wrangling, analysis and visualization were performed in R (version 4.2.2, R Core Team 2022) using tidyverse packages (dplyr, version 4.2.3, Wickham et al. 2023; tidyr, version 1.2.1, Wickham and Girlich 2022; ggplot2, version 3.5.0, Wickham 2016; ggpubr, version 0.5.0, Kassambara 2022; ggsignif, version 0.6.4, Ahlmann‐Eltze and Patil 2021).

## 3 Results

### 3.1 Performance

**FIGURE 1:**
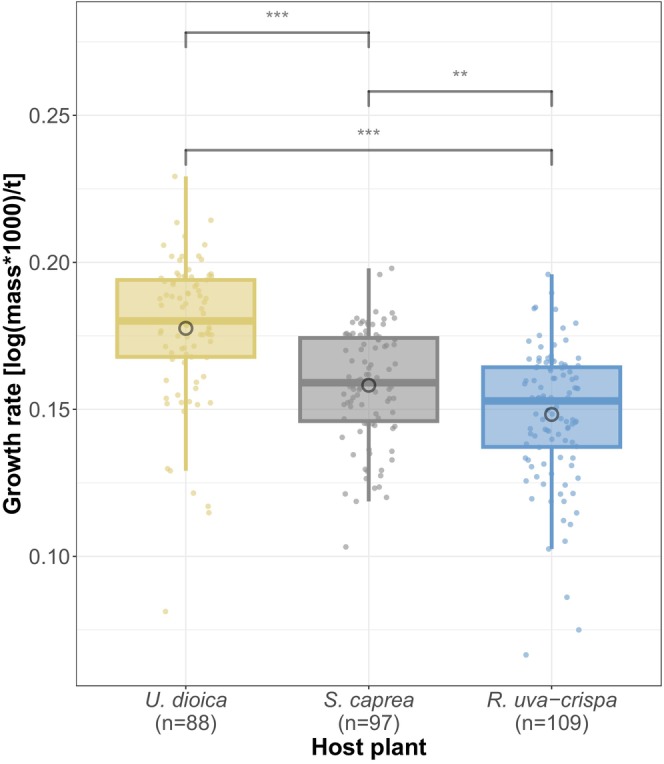
Host plant had an effect on the larval performance. Larvae were reared on either *U. dioica* , *S. caprea* or *R. uva‐crispa* . Upon reaching their 4^th^ instar, larvae were weighed and their growth rate was calculated using log(mass×1000)/developmental time. Boxplots show the median growth rate (center line), the 25^th^ and 75^th^ percentile (upper and lower box margins) as well as the 1.5× interquartile range (whiskers). Means are indicated by black circles within the boxes. Significance: ***p < 0.001, **p < 0.01; *p*‐values were extracted from post hoc tests from generalized linear mixed models. Sample sizes of measured larvae per host plant are reported on the x‐axis above.

Larval performance (i.e., growth rate) differed between the host plants (χ^2^ = 82.546, df = 2, *p* < 0.0001; Figure 1). As predicted, the highest growth rate was measured in caterpillars that fed on *U. dioica* , followed by those feeding on *S. caprea* and finally those on *R. uva‐crispa* . The high growth rate on *U. dioica* was driven by both the shortest developmental time and the highest mass at the 4^th^ instar (Figure S1).

### 3.2 Metabolic Rate

**FIGURE 2:**
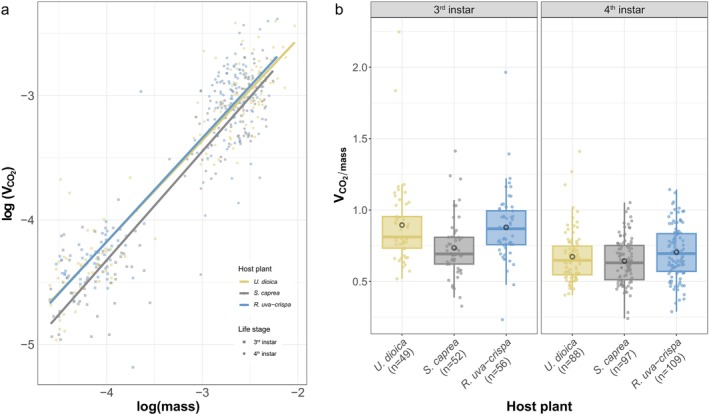
Diet dependent differences in the metabolic rate. The metabolic rate of individuals feeding on different host plants was assessed indirectly by measuring the amount of produced CO_2_. Given is the mass‐scaling (a) and mass corrected metabolic rate (b) of individuals reared on either of the three host plants. Note that division of the metabolic rate by mass was applied only for visualization. For both larval stages, boxplots show the median, the 25^th^ and 75^th^ percentile and the 1.5× interquartile range. Means are indicated by black circles. The number of measured individuals per group is given on the x‐axis below the host plants.

The metabolic rate of individuals feeding on different host plants was assessed at two developmental stages. The mass‐scaling exponent was 0.84 for all larvae regardless of host plant (χ^2^ = 2829.2, df = 1, *p* < 0.001; Figure 2), but the mass‐scaling coefficient differed among hosts with larvae feeding on *U. dioica* being 0.43, larvae feeding on *S. caprea* being 0.39, and larvae feeding on *R. uva‐crispa* being 0.44 (χ^2^ = 17.7, df = 2, *p* < 0.001). We could not find an effect of host plant in interaction with developmental stage (χ^2^ = 0.2444, df = 2, *p* = 0.8850), or developmental stage alone (χ^2^ = 0.3968, df = 1, *p* = 0.5287) on the metabolic rate. Host plant (χ^2^ = 15.6106, df = 2, *p* < 0.001) as well as logged larval mass (χ^2^ = 101.5543, df = 1, *p* < 0.0001) had a significant effect on the rate of CO_2_ production (Figure 2B). Pupae did not differ in their metabolic rate after feeding on different host plants (χ^2^ = 2.6895, df = 2, *p* = 0.2606; Figure S2). As the 4^th^ instar is the stage at which larvae begin intensive feeding, further analyzes focused only on this developmental stage.

### 3.3 Host Dependent Growth Costs

**FIGURE 3:**
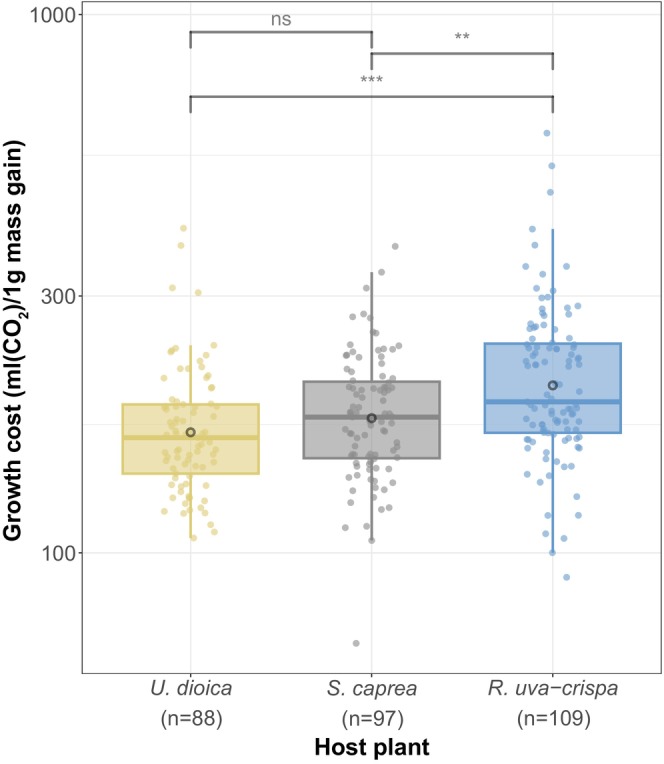
Growth costs differed between diet treatments in 4^th^ instar larvae. The cost for growth was calculated as the volume of CO_2_ produced for 1 g of mass gain. Larvae reared on *R. uva‐crispa* produced a significantly higher amount of CO_2_ per gram mass gain. Boxplots include median, 25^th^ and 75^th^ percentile as well as 1.5× interquartile range, and means (black circles). Significant differences between the diet treatments are shown by asterisks. *p*‐values were extracted from post hoc tests performed on generalized linear mixed models: ***p < 0.001, **p < 0.01, ns = not significant. Sample sizes are given below each host plant on the x‐axis. Note that the y‐axis is log10‐scaled for easier interpretation.

To estimate the amount of energy that is actually associated with the digestion of particular host plants, we quantified how much growth processes actually contribute to the metabolic rates described above. We found that the amount of CO_2_ produced for 1 g of mass gain differed significantly between the host plants (χ^2^ = 20.157, df = 2, *p* < 0.0001). While larvae reared on *U. dioica* and *S. caprea* showed similar host use expenses (*p* = 0.6396), individuals that feed on *R. uva‐crispa* showed a significantly higher cost per growth unit ( *R. uva‐crispa* vs. *U. dioica* : *p* < 0.001, *R. uva‐crispa* vs. *S. caprea* : *p* < 0.01; Figure 3).

## 4 Discussion

The individual performance of organisms can vary greatly depending on the prevailing environmental conditions. In the context of food, the availability of resources, but also their quality and composition, can have an important impact on an organism's survival, growth and reproduction. In this study, we provide an experimental framework in which we tried to identify the factors that underlie diet‐dependent performance differences. Combining performance assays, metabolic rate measurements and transcriptional profiles from earlier studies (Celorio‐Mancera et al. 2023; Schneider et al. 2024), we could show that differences in larval performance can, at least partly, be attributed to variation in energy allocation influenced by the composition of the consumed host plants.

In agreement with our first prediction, larval performance clearly differed between the different host plant treatments. The highest growth rate was measured on *U. dioica* , followed by *S. caprea* and the lowest growth rate on *R. uva‐crispa* . These patterns are in accordance with the results of earlier studies (Nylin 1988; Nylin and Janz 1993; Janz et al. 1994, 2009).

The energetic expenditure (metabolic rate) on the different host plants did not follow the patterns of the growth rate. This similarity in the metabolic rate between different diets agrees with previous studies that rejected the metabolic load hypothesis (e.g., Neal 1987; Castañeda et al. 2009). However, as mentioned in the introduction, the metabolic rate represents a measure of the total amount of energy required under certain conditions. The metabolic rate alone, therefore, only allows the conclusion that feeding on the different hosts is associated with comparable overall energetic costs.

The unraveling of energy allocation through growth costs provided more insight into the actual proportion of energy flow into digestion and related processes. With a comparatively high metabolic rate, *U. dioica* appeared to be a more “expensive” host than *S. caprea* . The high turnover rate (i.e., low amount of energy per 1 g mass gain), however, showed that a lot of this energy is directly invested into growth. This corresponds to the high growth rate measured on *U. dioica* . While showing a lower overall cost, larvae on *S. caprea* invested the same amount of energy into growth as on *U. dioica* . This seems to disagree with the growth rate differences measured between these two plants. However, it needs to be considered that larvae could follow different developmental strategies, where energy could either be invested into fast development or high end mass. Together with the differences we found in the developmental time, it seems that larvae reared on *U. dioica* direct the energy into fast tissue growth to complete their development quicker (cf. Janz et al. 1994; Nylin et al. 1996). This is also in agreement with the nutrient profile of *U. dioica* containing high levels of nitrogen (Taylor 2009). Increased levels of nitrogen have been shown to have a positive effect on developmental time, but also on individual mass (Nevo and Coll 2001; Huberty and Denno 2006; Rostami et al. 2012; Li et al. 2016; Lebigre et al. 2018; El‐Refaie et al. 2024). On *S. caprea* , in contrast, energy seems to be invested into the storage of resources, resulting in slower growth but heavier individuals (Janz et al. 1994; Nylin et al. 1996).

Notably, larvae reared on *R. uva‐crispa* required more energy for the same unit of growth compared to the other two host plants, indicating an increased energy demand for other processes. The transcriptional profiles described above provide good support that this energy is invested into detoxification of harmful substances (upregulation of Serine protease, Proline dehydrogenase, Aldoketoreduktase, Gluthatione‐S‐transferase 1 like) and coping with stressful conditions (upregulation of Spermine oxidase like, Esterase fe4 like; Celorio‐Mancera et al. 2013, 2023; Schneider et al. 2024). Here especially the response to oxidative stress (upregulation of Methione sulfoxide reductase) can be connected to the cyanogenic glucosides present in *Ribes*. Upon herbivore damage, these cyanogenic compounds can be hydrolyzed to hydrogen cyanide (HCN; Bjarnholt and Møller 2008). This reaction represents an immediate chemical defense mechanism, as hydrogen cyanide can obstruct cellular and physiological processes by binding on the metal ions in the functional group of different enzymes (see reviews by Boter and Diaz 2023; Krasuska et al. 2024 and references therein). Binding on the ion‐group in Cytochrome c‐oxidase, for instance, will increase the production of reactive oxygen species and can, thus, cause oxidative stress (Brattsten et al. 1983; and summarized by Boter and Diaz 2023 and Krasuska et al. 2024).

These coherences could offer good explanations for the different growth rates measured on different host plants, and especially the comparatively low growth rate on *R. uva‐crispa* . At a physiological level, this is furthermore supported by the lack of differences at the pupal stage. Diet‐dependent energy demands were measured shortly after the consumption of food, while pupae (that are not actively feeding anymore), did not show such responses (Figure S2). This suggests that differences in the energetic expenditure indeed arise from processes involved in the digestion of different hosts.

The results of this study indicate that the trend toward specialization could at least in part be driven by reduced energetic costs for detoxification and digestion of host plants and is, thus, in line with the metabolic load hypothesis. However, additional tests will be necessary to further validate this conclusion. In support of these results, increased specialization to *U. dioica* has been described. In more southern populations in Europe (e.g., United Kingdom, Belgium, Spain), populations of *Polygonia c‐album* are bi‐ to multivoltine, which means that they can produce multiple generations per year (Nylin 1988; Nylin et al. 2009). In these regions the higher costs and longer developmental time observed on *R. uva‐crispa* would have severe life history consequences. It is therefore necessary to use a host plant, like *U. dioica*, that enables fast development and, thus, ensures the completion of development in later generations (Nylin 1988; Nylin et al. 2009). In agreement, populations with more than one generation show a higher degree of specialization on *U. dioica* (Nylin 1988; Janz and Nylin 1997; Janz 1998; Nylin et al. 2009).

Importantly, several earlier studies have found quite opposing patterns, rather rejecting the idea of reduced performance due to higher energetic demands associated with detoxification (Neal 1987; Appel and Martin 1992; Van Loon 1993; Castañeda et al. 2009; Bastias et al. 2017). A possible explanation for the conflicting results could be that these studies used a measure for host use efficiency that only indirectly tested the actual energetic investment into different physiological processes. By using the dry mass of ingested and excreted food, they could get a rough idea of how much material was converted into new biomass (i.e., growth) and correlate this with the overall consumed oxygen/produced carbon dioxide.

In the present study we tried a novel approach, aiming to get a more direct estimate of energy allocation by calculating the growth turnover based on the amount of CO_2_ produced. Rather than inferring energetic investment from mass balance data (i.e., host use efficiency), this measure provides a more physiological estimate based on the actual metabolic rate and its consequences for growth. This allows for a more accurate assessment of differences in growth costs, which likely arise from host‐specific differences in digestion and detoxification.

Moreover, several of the previous studies used artificial diets with only the plant toxin varying in concentration (Neal 1987; Van Loon 1993). This is clearly a powerful approach if the aim is to study the causal effects of the toxins in separation. However, it is not only the chemical defenses themselves but also other host characteristics (e.g., leaf thickness, pubescence, accessibility of nutrients), that contribute to the overall digestibility (Matsuki and MacLean Jr. 1994). The metabolic efficiency of caterpillars of *Pieris brassicae* was, for instance, lower on actual host plants compared to an artificial diet (Van Loon 1993). An effect of structural traits (thickness) and primary chemical compounds on larval development and performance has also been shown (Wang et al. 2020). In addition, (host‐specific) endo‐ and epiphytic microorganisms could also trigger specific physiological responses and contribute to the digestibility of a host plant. The upregulation of genes involved in immune response including antimicrobial defense (Gloverin‐like protein, N‐acetylmuramoyl‐L‐alanine amidase, and Trypsin‐like serine protease), could indicate such a reaction to bacteria or fungi on *U. dioica* (Celorio‐Mancera et al. 2023). In contrast to artificial diets, the use of leaves therefore provides a more natural representation of the energetic costs associated with the processing of a host plant.

The timing for measuring the energetic expenditure could also be crucial. Assuming that detoxification processes and responses to hosts are activated shortly after feeding, most of the energy should be consumed during the actual digestion phase. We, therefore, conducted our measurements when we were sure the larvae had fed and we could expect a physiological response to the food. This is different from the approaches in some previous studies, where the assessment of metabolic rate ranged from measurements during the feeding process (Van Loon 1993) to ones in the post‐absorptive resting phase (Standard metabolic rate; Castañeda et al. 2009). Varying sampling timepoints could potentially explain varying conclusions about metabolic costs during the digestion of different diets. It should be noted that larval movement can cause variation in the metabolic rate (Nespolo et al. 2005). Larval activity was not recorded in this study; however, based on our observations in this and previous experiments, movement is strongly reduced in the absence of light. Although larvae were kept in darkness and did not show any visible activity during the incubation, we cannot fully exclude a minor contribution of movement to the metabolic rate. Since the incubation conditions were consistent across all developmental stages and host treatments, any residual activity would have affected all groups equally.

It should be noted that the approach used in this study provides only an overall estimate of the energy invested in digestion and detoxification within the measured time window of one hour. As a next step, future studies should incorporate flow‐through approaches to better compare the increase in metabolic rate after a meal (specific dynamic action; Smit et al. 2021). Examining differences in the shape and temporal course of this postprandial (i.e., post feeding) increase could further characterize the responses to more challenging hosts and provide deeper insight into the processing of different host plants. Furthermore, follow‐up experiments would benefit from more precise measurements of food intake. In the present study quantifying the amount of consumed leaf material was not feasible because leaves vary in structure and desiccation rates and weighing them before and after feeding could have introduced substantial measurement biases or would have required large amounts of additional leaf material. Differences in larval body mass, resulting from variation in feeding rate, may also reflect host‐specific behavior. Such behavioral responses are, however, part of food processing and therefore represent a relevant component of the host‐specific costs measured here. More detailed assessments of food intake could however help to further disentangle the factors contributing to these costs. In conclusion, we found that the differences in the larval performance on the host plants of *P. c‐album* could be explained by the metabolic load hypothesis. Although it was originally described in the context of tolerance against plant defenses, we propose that it should be extended to the overall composition of host plants. To give the entire environmental context of a food source it should therefore be considered to rather test metabolic costs on natural hosts rather than artificial diets, as this might also affect the ability and efficiency of detoxification and digestion. Connected to this, potential seasonal changes in the chemical composition and structural characteristics of a host should also be regarded.

Although our study focused only on *P. c‐album*, we believe that its diverse host repertoire with varying degrees of specialization to different hosts makes it a good model for understanding how host‐dependent metabolic costs can influence performance and, ultimately, the life history of herbivorous insects.

## Author Contributions

**Katharina Schneider:** conceptualization (equal), data curation (lead), formal analysis (lead), investigation (lead), methodology (equal), visualization (lead), writing – original draft (lead), writing – review and editing (lead). **Kevin T. Roberts:** conceptualization (equal), formal analysis (supporting), investigation (equal), methodology (equal), writing – original draft (supporting), writing – review and editing (supporting). **Philipp Lehmann:** conceptualization (equal), methodology (supporting), writing – original draft (supporting). **Christopher W. Wheat:** conceptualization (equal), writing – original draft (supporting). **Niklas Janz:** conceptualization (equal), writing – original draft (supporting). **Sören Nylin:** conceptualization (equal), funding acquisition (lead), resources (lead), supervision (lead), writing – original draft (supporting), writing – review and editing (supporting).

## Conflicts of Interest

The authors declare no conflicts of interest.

## Supporting information


**Appendix S1:** ece372509‐sup‐0001‐AppendixS1.docx.

## Data Availability

All data and relevant scripts for this study are provided at https://doi.org/10.5061/dryad.gb5mkkx42 and https://github.com/kschneider99/Pcalbum_MetabolicRate.
