# Supplementary material for: Quantitative Support for the Metabolic Load Hypothesis: Metabolic Rate Measures Reveal Host‐Dependent Growth Costs in a Polyphagous Herbivore
Source: Ecol Evol. 2025 Dec 11;15(12):e72509. doi: 10.1002/ece3.72509 (PMC12696725; doi:10.1002/ece3.72509)
Supplement: Supplementary file 1 — Appendix S1: ece372509‐sup‐0001‐AppendixS1.docx. [file ECE3-15-e72509-s001.docx]

**Supplementary Material**
**Quantitative support for the metabolic load hypothesis: metabolic rate measures reveal host-dependent growth costs in a polyphagous herbivore**

[Analysis of supplementary material 1](#_fgg6d5xen1h1)

[Figure S1. Mass and developmental time of 4^th^ instar larvae differed between host plants.](#_1zp3e8h0bbvz) 1

[Figure S2. Metabolic rate did not differ between pupae that were reared on different host plants. 2](#_j98vcpiw79io)

[References 2](#_psbsl4dwxd34)

#

# **Analysis of supplementary material**

The effect of host plant on the mass and developmental time of 4^th^ instar larvae (Figure S1) was tested with generalized linear mixed models using glmmTMB (version 1.1.9, Brooks *et al.* 2017) with host plant as a fixed effect. In both cases Family ID was included as a random effect:

Larval mass ~ Host plant + (1| Family ID), family = Gaussian

Developmental time ~ Host plant + (1| Family ID), family = Gaussian

To test for differences in pupal metabolic rate (Figure S2) connected to the diet, we tested for an effect of host plant on the CO_2_ produced. Logged pupal mass was included as another fixed effect. Again, Family ID was added as a random effect:

V̇CO_2_ ~ Host plant + log(Pupal mass) + (1| Family ID), family=Gaussian

Model fit was checked using DHARMa (version 0.4.6, Hartig 2022). Significance was assessed using the function Anova in the package car (version 3.1-2, Fox and Weisberg 2019).


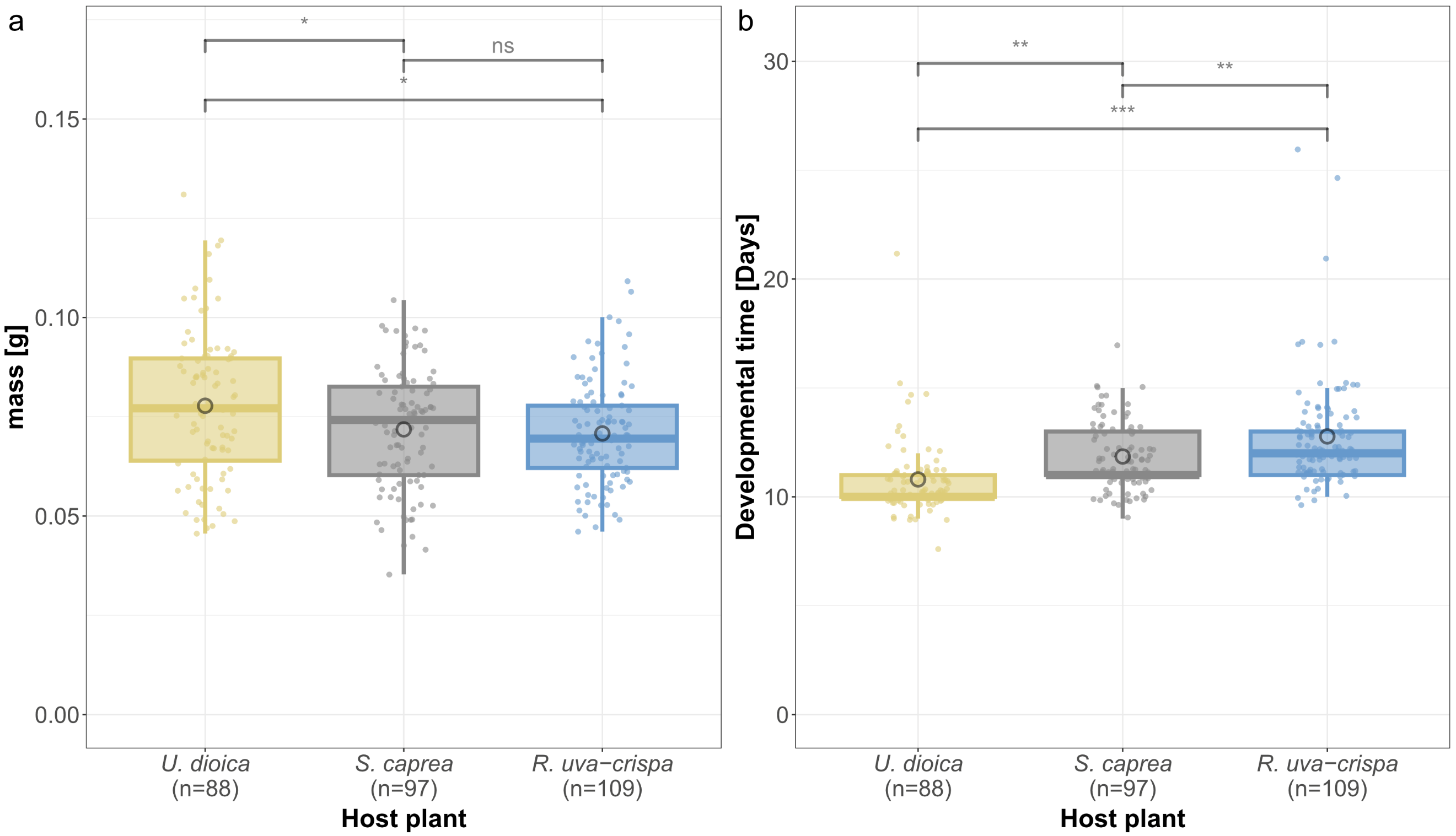


# **Figure S1. Mass and developmental time of 4^th^ instar larvae differed between host plants.**

Caterpillars that were reared on different host plants, were weighed upon reaching their 4^th^ larval instar (a). The time between hatching and reaching the 4^th^ instar was noted as developmental time (b). Boxplots in both panels show the median (center line), 25^th^ and 75^th^ percentile (box margins), 1.5x interquartile range (whiskers) as well as means (black circles). Significant differences between the diet treatments are indicated by asterisks: ****p* < 0.001, ***p* < 0.01, **p* <0.05, ns=not significant. *p*-values were extracted from post hoc tests performed on generalized linear mixed models.


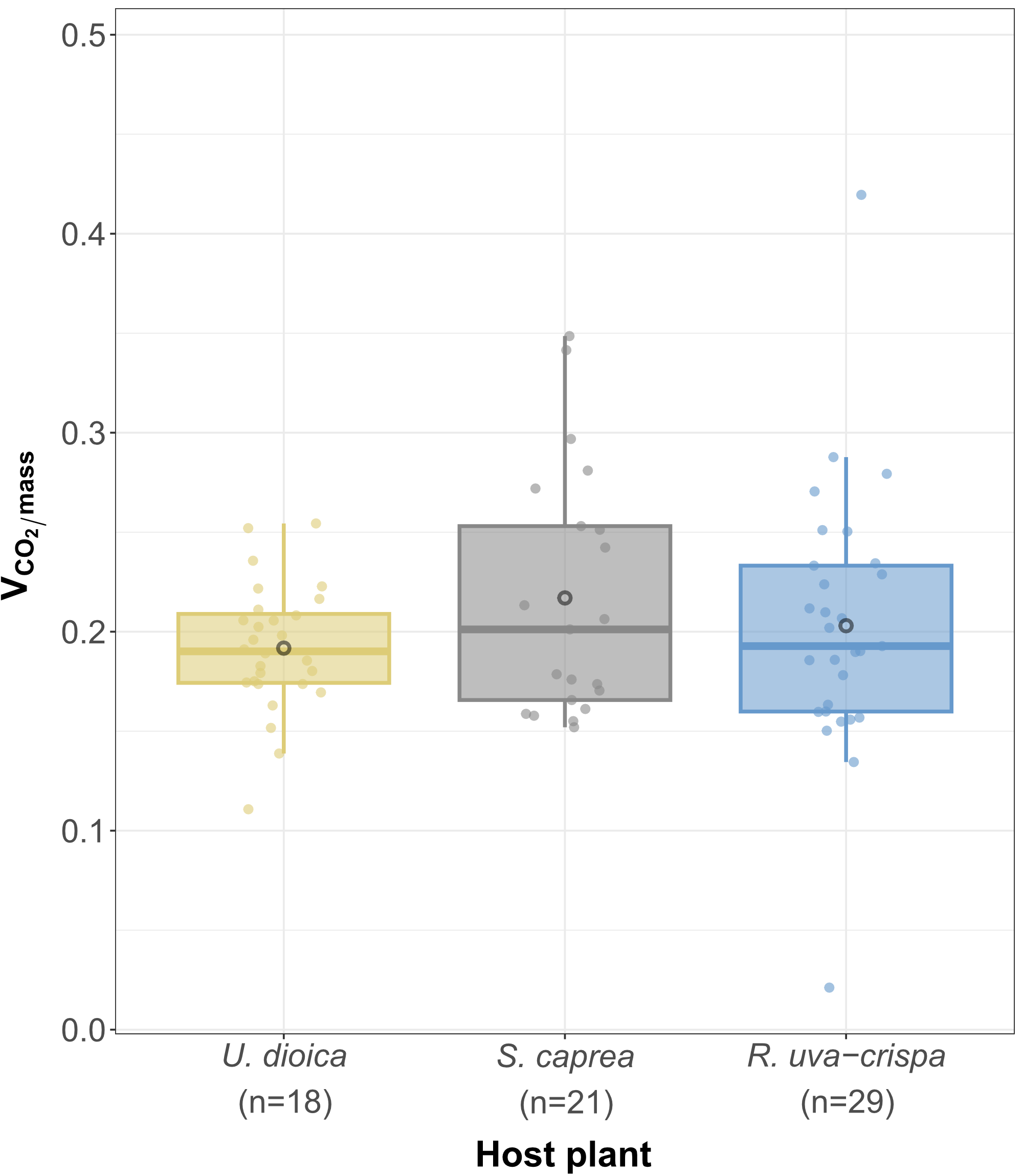


# **Figure S2. Metabolic rate did not differ between pupae that were reared on different host plants.**

The metabolic rate of pupa was measured between the 2^nd^ and 5^th^ day after pupation. Boxplots represent the median, mean (grey circles), 25^th^ and 75^th^ percentile, and 1.5x interquartile range.

# **References**

Brooks M E, Kristensen K, van Benthem K J *et al.* glmmTMB Balances Speed and Flexibility Among Packages for Zero-inflated Generalized Linear Mixed Modeling. *The R Journal* 2017;9:378.

Hartig F. _DHARMa: Residual Diagnostics for Hierarchical (Multi-Level /Mixed) Regression Models_.(R Package version 0.4.6 ) 2022. https://CRAN.R-project.org/package=DHARMa

Fox J, Weisberg S. _An R Companion to Applied Regression_. Sage (R package version 3.1-2) 2019.
